# Modes of Developmental Outgrowth and Shaping of a Craniofacial Bone in Zebrafish

**DOI:** 10.1371/journal.pone.0009475

**Published:** 2010-03-05

**Authors:** Charles B. Kimmel, April DeLaurier, Bonnie Ullmann, John Dowd, Marcie McFadden

**Affiliations:** Institute of Neuroscience, University of Oregon, Eugene, Oregon, United States of America; Harvard University, United States of America

## Abstract

The morphologies of individual bones are crucial for their functions within the skeleton, and vary markedly during evolution. Recent studies have begun to reveal the detailed molecular genetic pathways that underlie skeletal morphogenesis. On the other hand, understanding of the process of morphogenesis itself has not kept pace with the molecular work. We examined, through an extended period of development in zebrafish, how a prominent craniofacial bone, the opercle (Op), attains its adult morphology. Using high-resolution confocal imaging of the vitally stained Op in live larvae, we show that the bone initially appears as a simple linear spicule, or spur, with a characteristic position and orientation, and lined by osteoblasts that we visualize by transgenic labeling. The Op then undergoes a stereotyped sequence of shape transitions, most notably during the larval period occurring through three weeks postfertilization. New shapes arise, and the bone grows in size, as a consequence of anisotropic addition of new mineralized bone matrix along specific regions of the pre-existing bone surfaces. We find that two modes of matrix addition, spurs and veils, are primarily associated with change in shape, whereas a third mode, incremental banding, largely accounts for growth in size. Furthermore, morphometric analyses show that shape development and growth follow different trajectories, suggesting separate control of bone shape and size. New osteoblast arrangements are associated with new patterns of matrix outgrowth, and we propose that fine developmental regulation of osteoblast position is a critical determinant of the spatiotemporal pattern of morphogenesis.

## Introduction

Learning how different bones acquire unique morphologies has fascinated biologists for decades. Bone morphologies determine how the skeleton supports the soft tissues, how adjacent skeletal elements connect and function together, and the nature of muscular attachments to the skeleton. Since phylogenetically related vertebrates have skeletal systems made largely from the same (homologous) set of bones, the reshaping and resizing of individual bones is a critical consideration in the morphological evolution of new body forms.

Many investigations have addressed developmental mechanisms underlying skeletal element-specific shaping. The mesenchymal condensations from which skeletal elements arise have been singled out as being important for the patterning especially of chondral bones (i.e. bones developing out of a cartilage template (reviews: [1], [2]). Strong evidence for such condensation-intrinsic patterning comes from experiments where these condensations were placed into organ cultures, and observed to make cartilages with shapes reminiscent of those developing *in vivo* [3], [4]. Other signals likely come from outside the condensations themselves. Of particular interest for the shaping of intramembranous, or dermal bones that develop from mesenchyme not closely associated with cartilage, are the epithelia with which the skeletogenic mesenchyme is invariably associated. Grafting experiments point up the likelihood of signaling from such epithelia that initiates dermal bone development and may function in shaping as well [1], [3], [4]. Indeed, a suite of epithelial-mesenchymal signaling interactions, with signals going in both directions, likely underlies shape patterning of any dermal bone, as is currently under active investigation in many laboratories, including our own.

Furthermore, much is being learned at the molecular level about the signals and the responses of cells within the skeletal primordia to such signals. For example, a critical determinant of shaping of a set of chondral and dermal bones of the craniofacial skeleton is the signaling molecule Endothelin1 (Edn1), expressed by a ventral mesodermal core within the pharyngeal arches and ventral epithelia lining these arches. In response to Edn1 signaling, a variety of genes are transcriptionally upregulated in the neural crest-derived arch skeletogenic mesenchyme (reviews: [5]–[7]). The signal is transduced sequentially through a G-protein coupled receptor [8], [9], an associated signal-activation protein Phospholipase C-beta 3 [10], and a homeodomain transcription factor Mef2c [11], [12]. Transcriptional upregulation of selector Edn1-target genes follows, expression occurring in position-specific arch domains, controlled in part by interactions among the target genes themselves [13]–[15]. The new pattern of gene expression, in turn, determines the recruitment of regions of mesenchyme into skeletogenic lineages, i.e. it sets up a spatial fate map of the skeletal elements. Thus, at distinct dorsal-ventral positions within the posterior part of the second (hyoid) pharyngeal arch of the zebrafish, two early-developing dermal bones, the opercle (Op) and posterior branchiostegal ray (BR), sequentially ossify in the young larva. The more dorsal Op will form a plate-like rigid support of the gill cover, and BR will form a saber-shaped support of a more ventral and flexible region of the oral-pharyngeal cavity [16], [6]. Long before the stages of ossification, we are able to specifically identify the positions where the bones will appear as circumscribed domains of transcription of bone upstream regulatory proteins, including Runx2b, Osterix (Osx), and a number of others [17], [18]. In mutants with loss of function of the Edn1 pathway gene *furina*, dorsal-ventral patterning is misregulated and the Op-BR expression domains are expanded in size and frequently fused together, as revealed by expression of *runx2b* [17], the earliest of the osteogenic genes to be expressed [18]. This change in early positional specification of skeletogenic cells frequently results in a dramatic novel bone phenotype – the enlargement, reshaping, and fusion of the normally separate elements [19], [12].

In contrast to cartilage development (reviewed in [20]) understanding the course of bone morphogenesis – how the form (shape and size) of a bone is actually acquired – has not kept pace with the rapidly growing base of knowledge about molecular determinants of skeletal form. In part, this might be because the morphogenetic processes involved are complex and long in duration. A classic model for understanding dermal bone morphogenesis is the mandible of the mouse, with which a great deal of interesting work has been done [21], reviews: [22], [23], [1]. The adult form of the mandible is attained over an extended period that seems to involve cellular interactions of a number of types [24], including separate developmental modules [25]–[28] assembled from as many as five preskeletal condensations [21]. So far the studies have not revealed the specific natures of activities of the bone secreting cells, osteoblasts, during such extended development, or how these activities might differ during the formation of bones of different shapes.

Here we have used a model system approach to learn new features of bone morphogenesis. We examine shape development and correlated size increase (growth) of the zebrafish Op. Through a prolonged time-course of larval development we vitally stain the mineralized bone and image the living preparation at high resolution by confocal microscopy. This is a sensitive approach, essential for the detailed level of understanding we achieve. Staining the bone in strains in which osteoblasts transgenically express GFP informs meaningful interpretation of the cellular basis of morphogenesis. We show that the Op goes through major transitions in shape as it grows, and we use morphometrics to characterize apparently separate trajectories of shape development and growth. We observe that shaping occurs by differential outgrowths along the bone's different borders or edges, and a key finding is that distinctive modes of bone outgrowth – patterns of addition of new mineralized matrix – account for different aspects of morphogenesis. Two modes, spur and veil formation, produce new shapes with only minor increases in bone size. A third mode, leaving rings or ‘incremental bands’ [29] in the matrix, is the chief mechanism producing growth. Changing distributions of osteoblasts are congruent with the changing morphology, suggesting that the spatiotemporal patterning of osteoblast arrangement is a primary determinant of the trajectory of shape development. The complexity of the entire course of morphogenesis would seem to provide for many opportunities for regulatory controls that could be modified during evolution, therefore providing for outstanding diversity in bone morphologies.

## Results

### A Series of Shape Transitions Accompany Bone Growth

**Figure 1:**
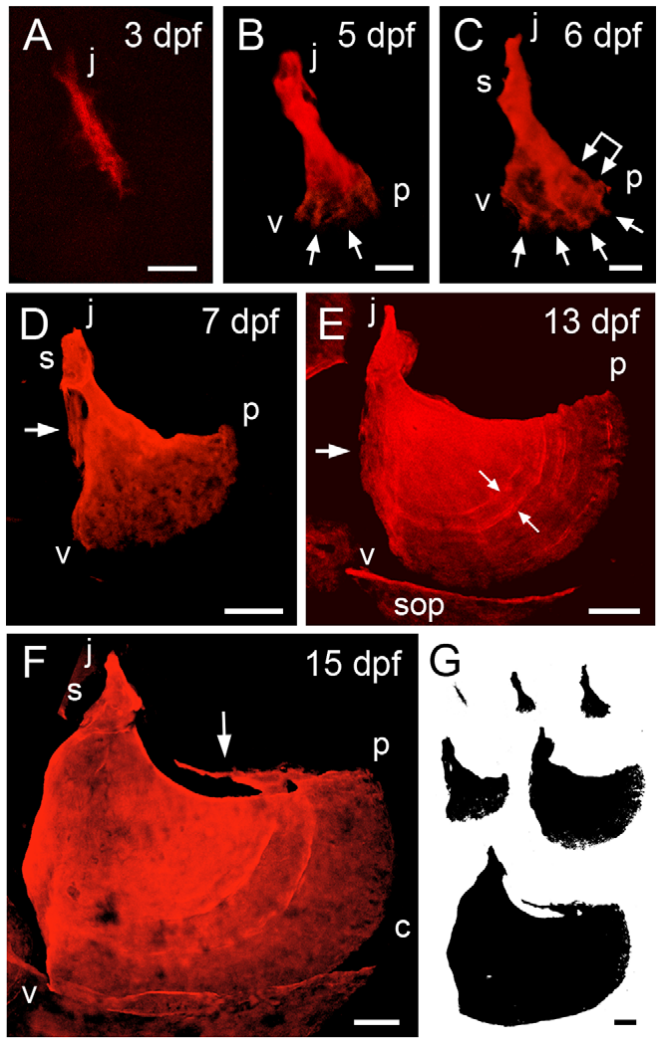
Time-course of shape changes and growth of the opercle in live, developing zebrafish larvae. A-F: Confocal projections made from z-stacks of images of live preparations vitally stained with Alizarin Red S. G: Silhouettes of the same bones scaled to the same final magnification to illustrate the amount of overall bone growth. Left-side views with dorsal approximately to the top and anterior to the left. The same orientations are used in all of the figures for this paper. (A) The Op initially ossifies as a linear bony spur. The more dorsal or j (‘joint’) end is adjacent to the hyosymplectic cartilage (not shown). In occasional preparations we first see the Op as just a spot of bone at what will become the j end. (B, C) Early fan-shaped Ops at 5 and 6 dpf, with three apices j (joint apex), v (ventral) and p (posterior). We use vj, vp, and jp to describe the bone edges between these apices. The j end of the element has elaborated a joint socket (s) component of the ball-and-socket articulation the Op makes with the hyosymplectic cartilage (for anatomy see [16]). The posterior-ventral end has broadened to form a new vp edge, by developing small, secondary spurs (arrows), with intervening thinly-mineralized veils. The linked arrow in C shows the first indication of incremental bands. (D) The fan shape is expanded at 7 dpf by differential elongation of the vp edge, relative to the other two. This vp elongation continues throughout the larval period. A new veil is evident along the vj (anterior) edge (arrow). (E) The vj veil is still evident at 13 dpf (arrow). A new dorsally pointing spur has appeared at the j apex, the site of attachment of the dilator operculi muscle. Incremental bands are visible in the matrix (e.g. at the small arrow pair). The image includes portions of neighboring ossifications present at this stage, including the subopercle (sop). (F) By 15 dpf curvature of the vp edge has locally increased at one region (c). The subopercle overlaps the Op vp edge ventrally. The dorsal jp edge, where the levator operculi muscle attaches, has developed a new veil. Scale bars: 20 µm in A-C, 50 µm in D-G.

**Figure 2:**
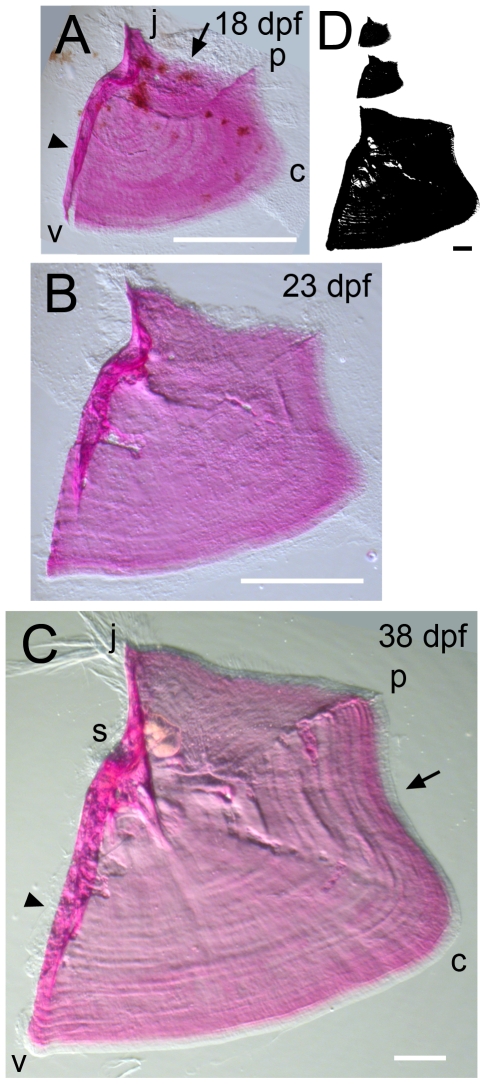
Time-course of shape changes and growth of the opercle in the late larva and in young adult fish. (A) 18 dpf. (B) 23 dpf. (C) 38 dpf. (D) Silhouettes scaled to the same final magnification to illustrate the amount of overall bone growth during this period. Imaging used oblique incident lighting to reveal matrix morphology. Incremental bands, the joint socket, and thickened, relatively heavily mineralized struts at characteristic positions along the bone are particularly well shown in C. The refractile fibers at the j apex in C are remains of the tendon that attached the dilator operculi muscle to the bone at this apex. Note a concave curvature along the vp edge (arrow in C) that was not present during the larval stages shown in Figure 1. Abbreviations and orientations as in Figure 1. Scale bars: 200 µm.

Craniofacial bones of the zebrafish, including the Op, undergo a remarkable set of shape changes as they develop from the time of first mineralization through the adult stage (Figures 1, 2). The Op is the first dermal bone to ossify in the pharyngeal arches [16], appearing at about 3 days postfertilization (dpf) when the embryo hatches to become a freely swimming larva. In such early larvae the Op usually has the form of a small linear ‘spur’ (Figure 1A), and at this stage we can recognize it only by its early time of formation and by its location, for neighboring craniofacial dermal bones also arise as linear spurs, each characterized by a specific time of appearance, location, and orientation (data not shown). In changes then specific for the Op itself, it develops a fan-shape with a joint socket at its upper end (anterior-dorsal, Figure 1B). The bone continues to change in form, such that within about another week, it looks in silhouette roughly like a duck with its beak pointing upward (Figure 1E). The Op in the young adult fish, in contrast, is approximately trapezoidal (Figure 2C). The scaled presentations in Figures 1G and 2D show the remarkable growth in bone size occurring all the while. We see striking allometry, meaning size-dependent change in shape, particularly throughout the period of larval development (approximately through 21 dpf), as we examine quantitatively further below.

We show below that matrix resorption and remodeling play no detectable role in shape development, at least during early stages. Because calcified bone matrix is rigid and nondeformable, all new growth has to occur at a pre-existing surface of the element, termed appositional growth [30]. Accordingly, change in shape can be understood as anisotropic matrix addition to pre-existing bone surface. Since the Op grows as a flattened plate-like element, most matrix addition will be at its edges. The outgrowth pattern we describe next, coming from interpretation of the nature of Alizarin Red staining of the mineralized matrix, receives support from sequential 2-color labeling of the matrix, and is further corroborated by observations of the changing distributions of osteoblasts, both studies described further below.

### Reshaping by Formation of Bone Spurs, Veils, and Bands

Looking in detail at the Alizarin Red labeling of multiple preparations at successive stages shows how different modes of outgrowth contribute to the bone's changing form. The early development of the fan shape involves a sort of branching morphogenesis, where, by 4 dpf, new secondary spurs of mineralized matrix develop along the outgrowing ventral-posterior end of the original spur (Figure 1B, C; arrows). The secondary spurs appear to increase successively in number and in length. However, this morphogenesis becomes more and more difficult to image at later stages as less densely stained small ‘veils’, sheet-like regions of bone, are deposited between the secondary spurs. Together, the spurs and intervening veils form a new, expanding ventral-posterior edge of the now triangular bone. For convenience of description we name the three apices of the triangle – j (joint), v (ventral) and p (posterior) – and name the edges as triangle sides jp, vp, and vj. In contrast to the activity at the vp edge, the vj and jp edges show little matrix addition at these earliest stages of ossification; hence just beyond the socket the element remains relatively thin. Growth along the vp edge broadens and lengthens the fan-shaped region of the bone. As early as 6–7 dpf we see, only in some preparations, hints of bands across the fan, near and parallel to the vp edge (Figure 1C, double arrow). We take this vaguely banded appearance (more prominent later; Figure 1E) as the first evidence of a third mode of matrix addition that previously has been described for teleost bones, incremental banding outgrowth. The vp edge is extended outward (i.e., away from the joint region in a posterior-ventral direction), relatively very rapidly and in a way that leaves bands in its wake [29].

During early morphogenesis, and due to the way the vp edge expands both outward and along its length (i.e., the length between the v and p apices), the Op takes on a form in which the rapidly growing vp edge curves outward in a convex manner and the two adjacent edges, vj and jp, show concave, inward curvatures. In what the matrix staining shows clearly to be a new phase of development, beginning at 7–8 dpf, a prominent veil of lightly mineralized new bone appears along the anteriorly located vj edge (Figure 1C, arrow). The veil is present at first near the upper part of the edge, near the joint. The veil expands ventrally (Figure 1E, arrow) and thickens (Figure 1F), such that the edge that was concave at first becomes convex. Then, near the end of larval development, the vj edge straightens out (Figure 2) by differential growth ventrally, and thickens markedly along its length, forming an edge-strengthening strut (Figure 2C).

A similar, but temporally independent phase of shaping occurs along the dorsally located jp edge of the Op, again prominently concave in the early larva and then straightening out. Here also, beginning at 15 dpf (substantially later than the vj veil just described), veils appear that are string-like at first (Figure 1F, arrow). The outgrowing edge then appears rather ragged (Figure 2A, B). As for the vj edge, a strengthening strut also develops, but comes to lie deep to the outgrowing edge, not along it (Figure 2).

In contrast to the broad vj and jp veils, an evident site of highly localized bone outgrowth occurs at the j apex, just dorsal to the joint socket. Here a new dorsally oriented spur appears, as early as 8 dpf in some individuals, invariably by 10 dpf. The spur, well shown in Figure 1E, elongates as a distinctive protuberance over the course of the next several days. It reaches maximal length, relative to the size of the whole bone itself, at 16–17 dpf, when outgrowth of the jp edge (as described just above) overtakes that of the j spur, such that the protrusion is relatively much shorter again (Figure 2). However, the spur remains evident as a line of heavy calcification, even after continued outgrowth in the adult.

### Local Matrix Addition and Not Remodeling Underlies the Shape Changes

**Figure 3:**
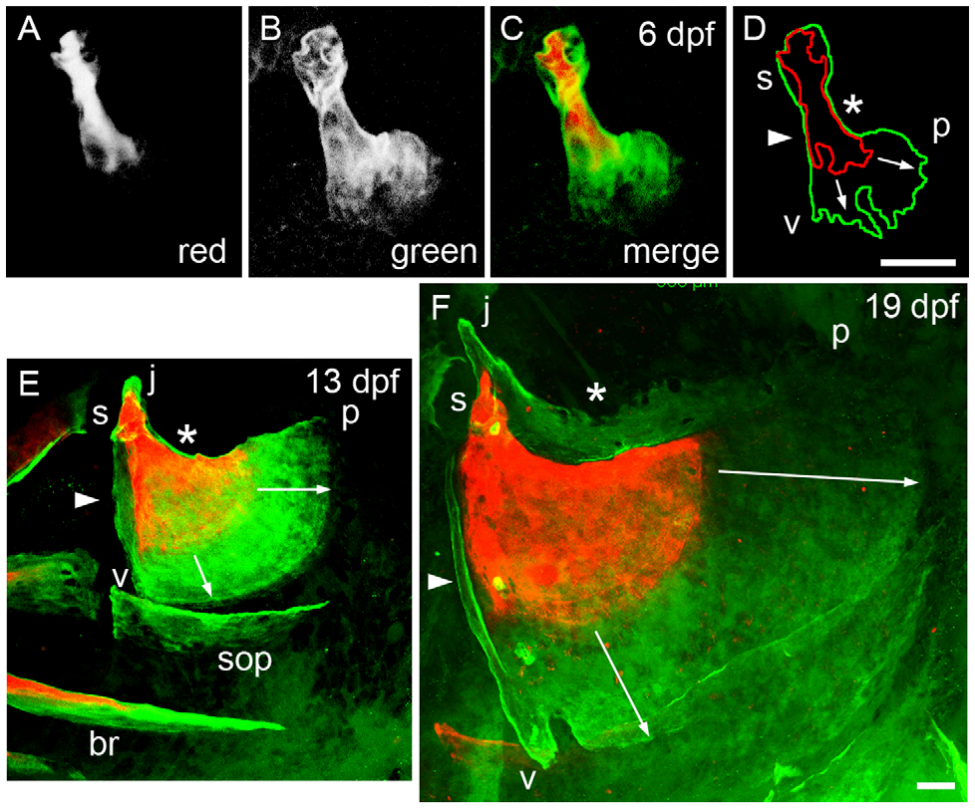
Mineralized matrix addition occurs in a stereotyped spatiotemporal pattern. Live confocal imaging as in Figure 1. The larvae were vitally stained successively with two Ca^2+^-binding dyes, first Alizarin Red S, then Calcein (green), with a washout period between the two applications. They were imaged just after the second staining period. (A-D) The same Op, stained first for 2 hr at 3 dpf (A, red channel), and then overnight between 5 and 6 dpf (B, green channel). C shows the merge and D the outlines of the two colors. The vj edge (arrowhead in D) and jp edge (asterisk) show only a thin layer of new (green) matrix on top of the older (red) matrix. In contrast, the vp edge, showing short spurs at the time of the first pulse, grows out prominently (arrows in D). (E) Another preparation in which a larva was stained with overnight first at 6–7 dpf and then at 12–13 dpf. The jp edge, as at the earlier stage, shows only a very thin layer of new (green) bone (asterisk). The veil along the vj edge (arrowhead), and the upward pointing short j apex spur are made of new bone. Note that this two-color matrix staining method also reveals that mineralization of the branchiostegal ray (br) began before 7 dpf (since the br is doubly labeled), but that subopercle (sop) mineralization is initiated only after day 7 (since the sop is not Alizarin Red-labeled). (F) A preparation stained overnight first at 12–13 dpf and then at 18–19 dpf. In contrast to the earlier stages, there is now an elaborate outgrowing veil, made of new bone, along dorsal jp edge. The anterior strut along the vj edge is new (arrowhead), and the j apex has elongated by new bone addition. Note that in both E and F the outgrowth of the vp edge is differential, more rapid near the p apex than near the v apex, as indicated by the lengths of the arrows. Abbreviations and orientations as in Figure 1. Scale bars: 40 µm.

In contrast to bone growth accompanied by incremental banding, we could not find published descriptions of the modes of spur formation and branching, and of veil formation. Early shaping might involve matrix remodeling, i.e., resorption by osteoclasts followed by new bone deposition in a different pattern. To test our interpretation that bands, veils, and spurs are revealing bone outgrowth and not remodeling, we carried out double-label experiments. We incubated the developing larvae successively with two calcium-binding dyes, Alizarin Red S and Calcein (green), with a period of washing out between the two applications, and we examined the two-color labeling pattern after the second application. An initial pulse of either dye will label the entire bone matrix present at the time of the pulse, and the bone-calcium binding appears stable such that little or none of the dye will transfer into new bone developing during the rinse period. A second pulse labels new bone Ca^2+^ that was not present at the time of the first, and hence not complexed to the original dye. This method confirms that the course of development of the initial fan-shaped region from a linear spur is accomplished by prominent new bone addition to a single end (the posterior-ventral end) of the spur present earlier. In the preparation shown in Figure 3A-D, from a spur with short branches labeled at 3 dpf (Figure 3A), there is specific elaboration a vp region by 6 dpf that is entirely new bone (Figure 3B, C, arrows in D). The joint socket (s) also enlarges. In contrast, in between the socket and the outgrowing vp region, the original spur only slightly thickens by addition of an extremely thin layer of new bone (arrowhead and asterisk in D).

Sequential double labeling also confirms that new bone addition forms the veil developing along the vj edge of the bone between 7 and 13 dpf (Figure 3E, arrowhead), the veils developing along the jp edge between 13 and 19 dpf (Figure 3F, asterisk), and the spur developing during this entire 12-day period at the j apex. Furthermore, older Alizarin Red S-stained bone has not been incorporated in the bony strut that develops after veil formation along the vj edge (Figure 3F, arrowhead).

### Incremental Bands

Once the early fan shape of Op develops such that the vp edge is present, the prominent mode of bone growth is the expansion outward, as well increase in length, of this vp edge. The outgrowth (Figure 3D-F, arrows) is accompanied by matrix banding, as noted above, and is ongoing. It clearly contributes in a major way to Op size development, while not so much to shape change, at least during early stages. We can consider the incremental bands to represent a historical record of the nature of outgrowth of the vp edge, fossilized in the matrix behind this edge. This developmental history is useful for understanding how the Op is growing in size and, later, how it reshapes as it grows. In the larva we only sometimes can visualize the bands, so the record is an imperfect one, but our data are suggestive that the bands often record daily growth increments. For example, the band widths in the 13 dpf preparation shown Figure 1E are about 10 µm near the v apex and 14 µm near the p apex. Daily outgrowth of the vp edge measured between 7 and 13 dpf in 3 preparations including the one shown in Figure 3E is 10.0 µm/day near the v apex and 16.5 µm/day near the p apex.

Outgrowth rate is higher in the older larvae. We measured this outgrowth in 3 preparations between 13 and 19 dpf, including the one in Figure 1F, and observed rates of 21.6 µm/day near the v apex and 32.0 µm/day near the p apex. The bands near the bone edge are wider in the older larvae as well (Figure 3F vs. E). These data suggest that rates of incremental banding outgrowth are regulated spatially along the length of the vp edge as well as temporally in a stage-dependent fashion. This differential outgrowth likely underlies the reshaping of the vp edge that occurs during this period of larval development. Between 4 and 11 dpf the convex curvature along the edge is fairly uniform. Then the edge straightens out near the v apex, and remains prominently curving near the p apex (Figures 1F, 3F), where we observe the relatively faster growth and larger bandwidths. At postlarval stages (ca. 21 dpf and later), we see a new pattern where the bands are widest at the prominent convex curvature along the vp edge (“c” in Figure 2C), and narrowest in the region between c and p. This zone of narrow band widths showing reduced local outgrowth coincides with where a prominent concavity develops along the bone edge, beginning at 18–20 dpf, as is evident in Figure 2.

**Figure 4:**
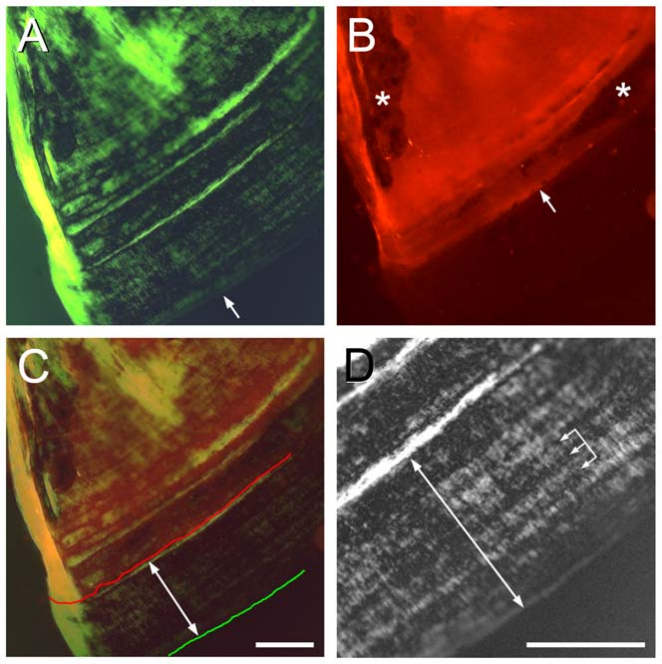
The Op of the young adult shows a high rate of incremental banding outgrowth of the vp edge. (A) Image at 54 dpf with green monochromatic (to increase resolution) transmitted light, and crossed-polarizing filters to reveal birefringence and the incremental banding pattern. The arrow indicates the vp edge of the bone. (B) Epiflorescence of Alizarin Red S, applied as a pulse at 43 dpf, in the same field as in A. The labeling front (arrow) shows where the vp edge was located at the 43 dpf stage. Sites (Howship's lacunae) of likely remodeling (bone resorption by osteoclasts, followed by replacement with new, unlabeled bone) in the old bone behind this front are indicated by asterisks. (C) Merge of A and B. The Alizarin Red front is indicated by the red line, the bone vp edge by the green line, and the double-headed arrow shows the approximate extent of outgrowth during the 11 day interval after labeling (an average 333 µm, from several measurements along the bone). (D) Detail of the banding pattern between the labeling front and the bone edge (double-headed arrow). Widths of prominent bands are about 30 µm; the three linked arrows show two 30 µm intervals. We note more finely spaced bands are also present, and that we measured a substantial variation in bandwidths among different preparations. Scale bars: 200 µm.

The Op growing in the young adult maintains a high rate of vp incremental banding outgrowth, higher than in the larva. In one set of young adults the Op was vitally stained with Alizarin Red S at 43 dpf, followed by an 11-day period with no stain present, and imaged at 54 dpf (Figure 4). Outgrowth during the 43–54 dpf interval in the example shown, measured from the region of unstained bone near the v apex amounted to 333 µm (Figure 4C, D; double-headed arrows), an average of 30.3 µm/day. The banding pattern was imaged by its birefringence (Figure 4A, D) and prominent bands were present in this same region of newly-added bone with an average spacing of about 30 µm.

The issue of daily increments of addition of bone laminae is complicated by the complexity of the banding pattern; individual bands can merge together, as described by Smolyar for salmonids [29]. Sometimes the bands appear doubled, and in some preparations we could image fine bands occurring in the intervals between the larger ones, a few examples are present in Figure 4D. This complexity suggests that a circadian basis to the incremental bands, assuming one to be present, is not always strict.

We note an example where incremental bands appear along a new edge rather late in development. At first, banding is restricted to the vp edge. In contrast, the early jp edge, as described above, expands outward by addition of veils. However, after this initial outgrowth of the jp edge, it later shows bands (revealed by birefringence in young adult fish, data not shown). However, we never observed bands along the vj edge. Additionally at postlarval stages we see evidence of active bone resorption, both using vital labeling (Figure 4B, *), and by visualizing matrix depressions with the appearances of Howship's lacunae [30]. We have not observed such regions during larval stages.

### Trajectory of Op Morphological Development

The Op undergoes a series of shape transitions, underlain by distinctive modes of bone matrix addition, as it enlarges dramatically in overall size through an extended course of development. Using methods of geometric morphometrics we quantify the changes in both shape and size (centroid size, CS), to learn how they may correlate with one another, as well as with developmental age. We use as shape traits the first two principal components (PCs) deriving from this analysis, PC1 and PC2, which together explain 88% of the total shape variation in our dataset.

**Figure 5:**
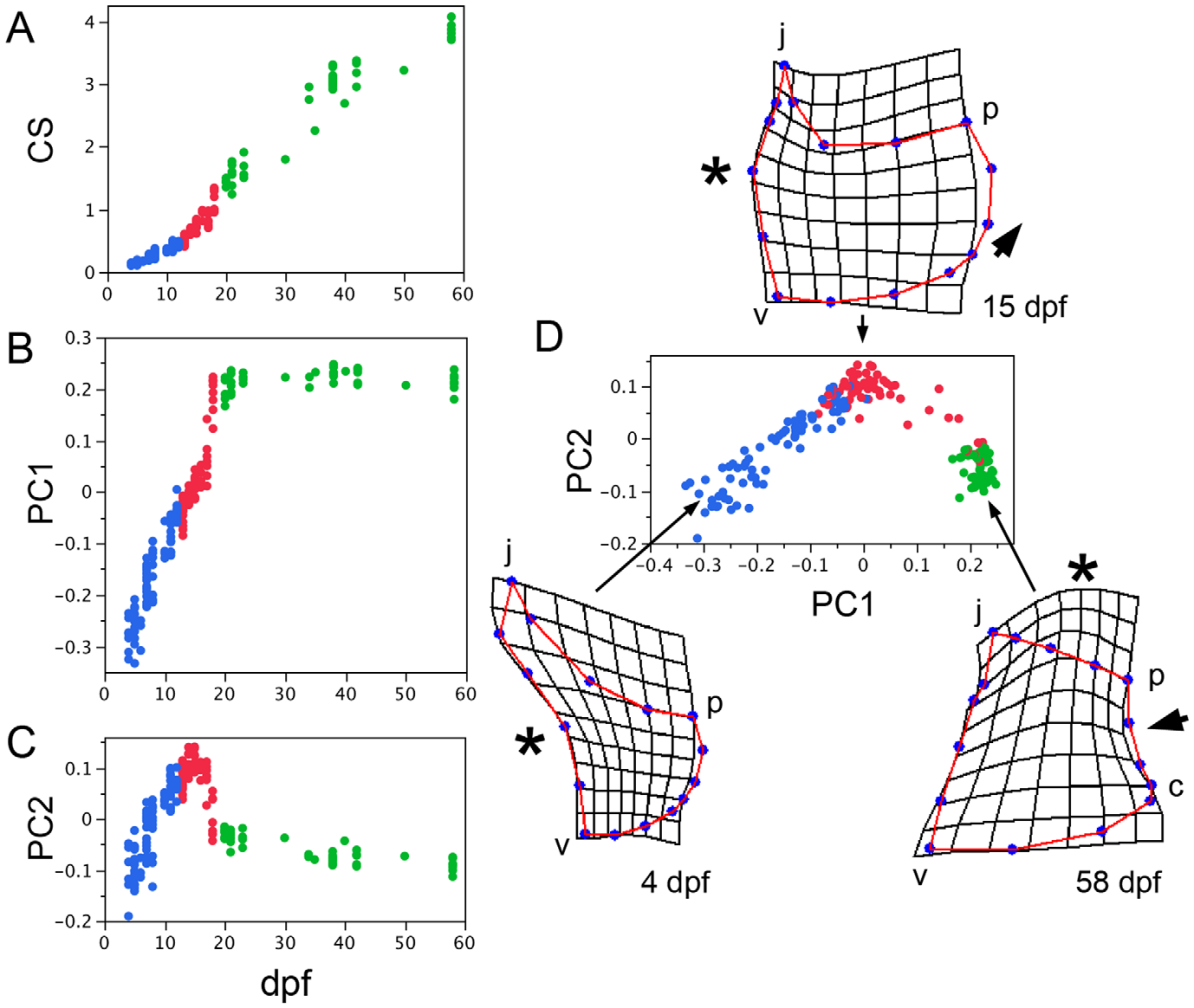
Morphometric analysis of opercle morphogenesis between 4 and 58 dpf reveals separate nonlinear trajectories of growth (A) and shape change (B-D). The data points in the bivariate plots indicate individual samples, color-coded by age: blue, 4–12 dpf, red, 13–18 dpf, green, 20–58 dpf. (A) Centroid size (CS) versus dpf. (B, C) Shape summary variables PC1 and PC2 versus dpf. (D) PC1 versus PC2. PC1 explains 73% and PC2 explains 15% of the total shape variation in the dataset (n = 176). The diagrams that accompany the bivariate plot in D show the Op shapes at the indicated stages as average thin plate spline deformations from the consensus configuration for the entire dataset (not shown). For the 4 dpf average n = 7, for 15 dpf n =  10, and for 58 dpf n =  7. Asterisks and large arrowheads point out prominent developmental changes described in the main text.

Bivariate plots of CS and the two shape traits, PC1 and PC2, with developmental age (dpf) each show distinctively shaped distributions (Figure 5A-C). Increase in CS follows an S-shaped curve, which in fact appears linear over an extended course of development, from about 8 through 40 dpf (Figure 5A, slope = 0.098 for this portion of the curve, R^2^ = 0.97). In control experiments not shown we determined that CS is proportional to the square root of the bone's surface area, meaning that the bone is adding surface at a rate proportional to CS^2^. We also found, in a subset of fish for which we have standard length (SL) data, that CS is strictly proportional to body size; CS regressed on SL yielded a straight line (CS = −0.5+0.17SL, R^2^ =  0.99, standard error = 0.001, n = 28).

The shape traits PC1 and PC2 show quite different developmental trajectories from CS (Figure 5B, C), suggesting that developmental regulation of shape and overall size are at least partially independent. The PC1 and PC2 trajectories differ from one another as well. PC1 rises approximately linearly throughout larval development (slope = 0.029 during the rising phase, R^2^ = 0.91) and reaches a plateau at about 20 dpf (Figure 5B, stages from 20 dpf onward are color-coded green), which is close to the time of the larval/juvenile transition [31]. PC2 also rises at first (4–12 dpf data color-coded blue) but then reaches a maximum at 13–14 dpf, and declines rapidly (13–18 dpf data colored red). The rate of decline then slows considerably, the transition from the rapid to the slow decline closely matching the onset of the PC1 plateau (green points).

By plotting PC2 versus PC1, as shown in Figure 5D, we can generate a pure bivariate “shape space” and show the developmental trajectory through this space. This plot emphasizes that by far the greatest change in Op shape is occurring during larval development, because the green points, representing postlarval stages, are closely bunched together. Furthermore, it is clear that at day 4 and afterwards during the larval period, shape development is predominantly occurring as two phases, an early rising phase followed by a falling phase, the difference between the two phases dominated by PC2.

A tool provided by the tpsRelW software that we used for this analysis allows one to visualize the shape deformations associated with particular PC scores. We illustrate these shape changes (in Figure 5D) as thin plate spline grid deformations of the consensus configuration (where PC1 and PC2 both equal 0) superimposed on a rectangular grid, in the style made famous by D'arcy Thompson [32]. With deformation toward negative values for both PC1 and PC2, where the early larval form maps on the space, we see that the left part of the grid is notably compressed (asterisk at 4 dpf, Figure 5D). The rise to high PC2, where the midlarval form maps on the space, is a relaxing of this compression and the appearance of an outward, or convex bulge along the grid's upper left region (asterisk at 15 dpf). The change can be related in a straightforward way to our analyses of the bone staining patterns described above; it corresponds to veil formation and then outgrowth along the bone's vj edge. The 15 dpf grid also shows, at the upper left, the outgrowth of the j apex spur and the elongation of the vp (large arrow). During the subsequent falling phase of the developmental trajectory a prominent change occurs in the uppermost region of the grid; it becomes prominently convex (asterisk at 58 dpf). In contrast, a convex curvature on the right side of the grid is reversed to a concave one (large arrow at 58 dpf). Both of these changes also have their biological correlates; respectively, the vp veil development, and the locally reduced rate of banding outgrowth (between regions p and c).

It is notable that all of the stage- and region-specific modes of outgrowth we found in our study of the bone's staining patterns, and that we interpreted to underlie specific shape developments, show up as well in the thin-plate spline analysis. Hence the biological and statistical analyses agree with one another. Significantly, there is no bulging outward to the grid's lower right, that one might have expected because of very prominent incremental banding outgrowth in this direction of the vp edge described above. We interpreted this outgrowth as contributing primarily to the increase in size of the Op, but not changing its shape very much. Again, the two kinds of analyses would seem to be in agreement.

### Changing Osteoblast Distributions Correlate with the Changing Patterns of Bone Outgrowth

**Figure 6:**
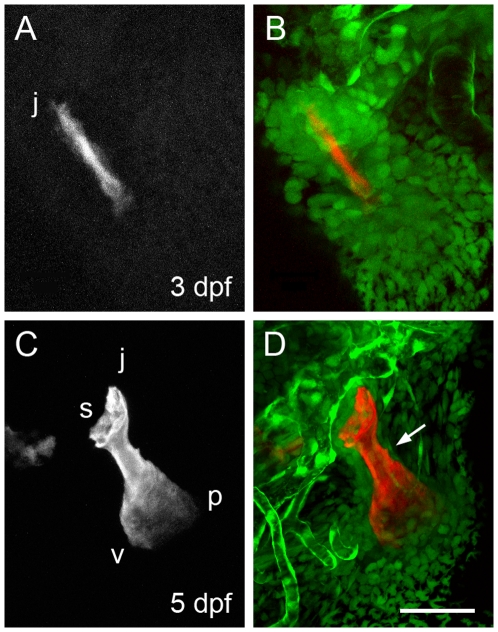
Arrangements of neural crest-derived mesenchymal cells associated with the opercle developing in the young larva. Two-color confocal imaging of live preparations. (A, C) Red channel at 3 and 5 dpf showing the Alizarin Red S labeled bone. (B, D) Merge of the red channel and the green channel showing cells expressing the *fli1:eGFP* transgene. Endothelial cells of capillary tubules also brightly express this transgene. The dense condensation of Op-associated cells present at 3 dpf thins out considerably by 5 dpf, particularly along the very slowing growing jp edge of the bone (arrow in D). Abbreviations and orientations as in Figure 1. Scale bar: 50 µm.

**Figure 7:**
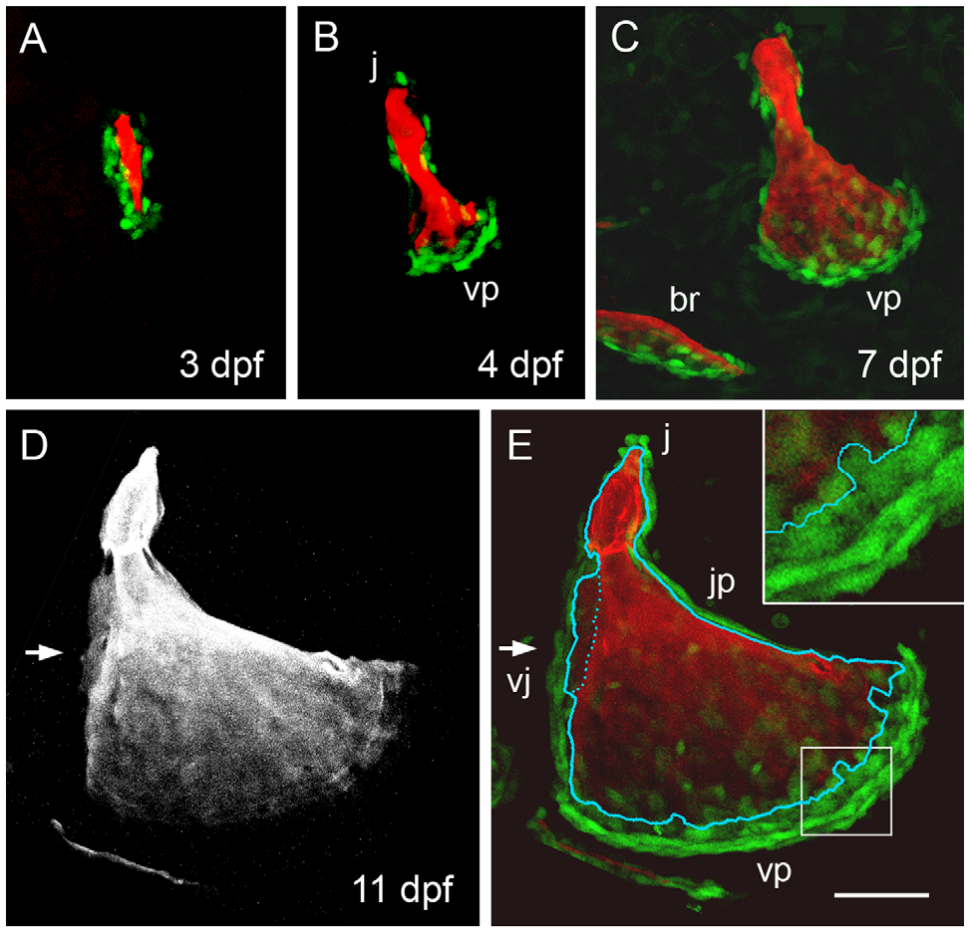
Osteoblast arrangements change dynamically during opercle morphogenesis. Confocal imaging of live preparations. (A-C) Two-color merged images showing Alizarin Red A labeling of the matrix and *osx*:eGFP labeling of the bone forming cells. (A) At 3 dpf osteoblasts line up along the developing bony spur. (B, C) At 4 and 7 dpf a new arrangement is present with cells especially concentrated along the rapidly outgrowing vp edge. The newly forming posterior branchiostegal ray (br) is included in C. (D) Red channel, and (E) merge at 11 dpf. Arrows indicate the vj veil. In E the outline of the Alizarin Red labeled bone (from D) is superimposed. Very flattened and compact looking *osx:eGFP*-expressing cells line the very slowly growing jp edge and are present in outer rows of the very rapidly growing vp edge. Small round cells are present at the j apex spur. Larger diffusely labeled cells are present along the vj veil and in the innermost row along the vp edge, where the cells immediately contact new mineralized matrix (a portion of this edge is enlarged in the inset). Scale bar: 50 µm.

How osteoblasts develop and regulate their activities is responsible for the different modes of bone outgrowth we have described. To learn about their distributions, and as well, the distributions of their putative precursor cells in the skeletal mesenchyme associated with the Op, we examined Alizarin Red labeling in two strains of eGFP-expressing transgenic zebrafish. Imaging fish carrying the *Tg(fli1a:EGFP)y1* transgene (abbreviated here to *fli1:eGFP*) allows us to visualize possibly all cranial neural crest-derived mesenchyme (Figure 6). By imaging fish bearing the *Tg(osx:egfp)b1212* transgene (*osx:eGFP*), the osteoblasts are revealed highly selectively (Figure 7).

As shown by *fli1:eGFP* expression, a dense condensation of crest-derived mesenchyme surrounds the newly mineralizing bone at 3 dpf (Figure 6A, B). The condensation consists of densely packed cells, and appears clearly associated with the developing bone, even though it may include cells other than Op-forming osteoblasts and their precursors. Cells in the surrounding mesenchyme outside of this cloud are much more loosely associated, and more irregularly shaped. Within the next few days the Op-associated mesenchyme thins out considerably over the flat faces of the developing bone and along its length (e.g. especially along the jp edge, arrow in Figure 6D). In contrast, cells remain accumulated in the joint region, and particularly along the vp edge, which is covered by a multilayer of expressing cells. These distributions are suggestive that the mesenchyme reorganizes in a way that directly reflects the new patterning of outgrowth.

This proposal of cellular redistribution is supported by inspection of the arrangements of *osx:eGFP*-expressing cells, clearly much more selective for active bone-secreting osteoblasts (Figure 7). The labeling pattern in the transgenic line is comparable to cell labeling by *osx*-mRNA in situ hybridization ([18], DeLaurier, in preparation, and data not shown), suggesting that that we are not missing *osx*-expressing cells by using the transgene. Around the time of initial ossification, the osteoblasts line up along the growing spur (Figure 7A). Thereafter, the largest numbers of *osx:eGFP* expressing cells are closely associated with the rapidly growing vp edge (Figure 7B, C, E). Later, new local accumulations of cells accompany spur formation at the j apex (Figure 7E), vj veil formation (Figure 7D,E, arrow), and still later during jp veil formation (data not shown)

Close inspection of cell-bone matrix association in Figure 7E reveals that *osx:eGFP* expressing cells are closely associated with the mineralized matrix edge irrespective of whether the matrix is locally growing rapidly or very slowly, but are fewer in number and highly flattened in shape along the slowly growing edge (jp). The cells are in layers along the vp edge that exhibits the most rapid outgrowth. Here the innermost cells at this edge are immediately adjacent the mineralized matrix edge, and these cells are large and rounded whereas cells in the outer layers are more elongated and compact looking (inset in Figure 7E). It would seem likely that the dense and layered cellular arrangement at this location somehow specifically and directly accounts for the extremely rapid banding mode of outgrowth at this edge.

## Discussion

### Growth Allometry

Op shape development occurring in the zebrafish larva is characterized by marked allometry – change in form accompanying growth. Early mineralized matrix, appearing around the time of hatching of the embryo (3 dpf) is in the form a linear spur, a form that we noted to be shared with other craniofacial bones of dermal origin. The spur develops within a condensation of neural crest-derived cells, and we interpret a subset of these cells immediately associated with the spur to be active matrix-secreting osteoblasts, as indicated by their morphologies and especially by their expression of eGFP driven by *osx*, a gene encoding an upstream bone regulatory transcription factor. The early Op then undergoes a characteristic and stereotypical sequence of morphological change as it grows, especially pronounced during larval development. Osteoblast distributions change as well, and their increased numbers at sites of new bone outgrowths suggest that their arrangements are the critical determinants of the changes in bone shape. Approximately at the larval/juvenile transition (21 dpf; [31]), shape change slows down abruptly and very considerably, while the bone continues to grow in size over the 60-day period that we followed it. This later growth occurs without nearly so much accompanying shape change as earlier, and (as also during larval development) is closely coupled to growth of body size of the fish. Multivariate analysis revealed that different trajectories describe bone size and shape development. This finding was surprising, given that our data clearly show that new shapes arise as new matrix outgrowths, thus size increase certainly accompanies allometric reshaping. We suggest that the reason the size and shape developmental trajectories look so different is because size increase of the Op, throughout its entire course of development, is dominated by a single mode of outgrowth – incremental banding growth of the vp edge. New shapes notably arise by other modes at other bone edges, which, even though crucial for reshaping, contribute in a relatively minor way to overall bone size. This point is corroborated by thin-plate spline analysis and dramatically illustrated in our 2 color matrix-labeling experiments; e.g. compare the size of the green staining vj veil to the much larger size of the green staining vp region that grows out by incremental banding growth in Figure 3E. Outgrowth of both regions occurred during the same six day interval.

We note that we have only described bone-forming cells, osteoblasts, as being present along the bone surfaces. We also see, in the young adult, a sparse population of ostocytes in the central regions of the Op – cells fully encapsulated in lacunae within the matrix. This condition is characteristic of other basal teleosts, whereas “acellular bone” in the adult is a derived state present in some teleost lineages [33]. We have not systematically examined when and how the osteocytes appear during development.

### Modes of Bone Outgrowth

Incremental bands in the matrix of teleost bone, particularly scales, is well known to fisheries biologists, who examine the banding patterns to determine the age of a fish, and conditions of the environment in which it lived [29]. The bands themselves may well be one and the same, biophysically, with bone ‘lamellae’, birefringent bands that characterize the remodeled bone of Haversian systems in birds and mammals, and that depend ultimately on the submicroscopic organization of the matrix [34]. The cellular behaviors that generate bands are unknown. The zebrafish bands form more rapidly than described for mammalian bone lamellae (a band of 30 µm growing during about one day for the zebrafish adult Op, vs. lamellae of about 5 µm width growing over the course of about 5 days in the mammal [35]. The rate of incremental banding outgrowth largely determines the rate of growth of the bone itself, and different local regions of the Op vp edge show different band widths (e.g. highest at the location ‘c’ in late larvae and afterward), such that banding contributes to bone shaping as well as growth in size.

In addition to incremental band formation, we described two modes of outgrowth that generate new bone shapes, spurs and veils. Formation of both small spurs and intervening small veils seem to generate the early flat, triangular shape of the Op, and in later morphogenesis veils and spurs develop at specific stages and locations. Spurs are linear outgrowths that elongate by focal growth of their ends (e.g. as for the primary spur at 3 dpf, and the j apex spur later). In contrast, veils appear diffuse and lightly mineralized when they first appear and they also enlarge in a rather diffuse fashion. The light mineralization is transient; there is no hint, looking at the Op at a late stage, where veils were present earlier. The importance of the veils in shaping is pointed up by our geometric morphometric analysis. This analysis revealed that after 4 dpf when the bone is triangular in shape, subsequent shaping has two major phases. Veil development, first along the vj edge and then along the jp edge, contributes markedly to each phase.

**Figure 8:**
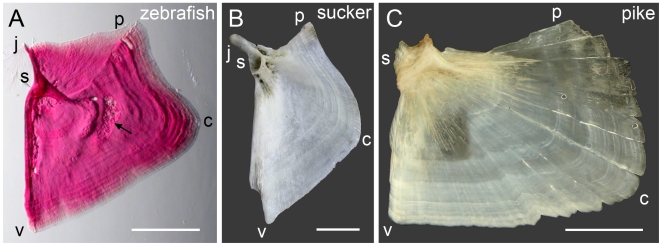
Comparison of opercle shapes of (A) zebrafish, *Danio rerio*, (B) sucker, *Castostomus sp*., and (C) northern pike, *Esox lucius.* Letters along the edges indicate hypothetically homologous locations along the bones. Shape differences along the upper (jp) edge and the j apex are discussed in the text. The arrow in (A) indicates a prominent Howship's lacunae, a site of osteoclast-mediated bone resorption. The vp edge appears to outgrow differentially in all three species, as indicated by the incremental bands being wider in region c. The region between c and p shows the narrowest banding for both zebrafish and sucker, resulting in the concavity here shared by these two species but not by the pike. Zebrafish and the sucker are placed in separate families within the order Cypriniformes, the pike is in the order Esociformes and therefore is an outgroup to the other two species. Scale bars: 0.5 mm (A), 10 mm (B,C).

The time- and position-specific appearances of spurs and veils motivate an hypothesis that modes are regulated independently from one another in the fashion of developmental modules [36]–[38], [28]. We further propose that modification of development of the modes, individually, is a way to effect evolutionary change in Op shape, which in fact varies markedly among teleosts [39]. Modularity could serve to produce independent changes in outgrowth along the different edges of the Op, and hence account for its apparent highly evolvable shape. For example, the Op in suckers (*Castostomus sp*.) has a prominent concave curvature along its jp edge [40], rather than a straight edge as in the adult zebrafish (Figure 8A, B). This jp curvature is present early in sucker development [41], matching zebrafish early larval stages, and the straightening of the jp edge out by the veil that we showed then develops in zebrafish may well have been skipped over in the sucker. A similar case can be made for the northern pike (*Esox lucius,* Figure 8C). Furthermore, the pike has no prominent extension dorsal to the joint, which would correspond to the j apex of the zebrafish Op, and remains a prominent protuberance in the adult sucker. We can suppose that the extension in the sucker develops just as in zebrafish – from a j-apex spur, but that the spur never forms during development of the pike Op (an extension is not figured in a developmental study of the pike [42]). These examples are just two among many in teleosts that suggest modular regulation of the developmental modes we have described, as could be critically examined by looking more closely at the appropriate stages of the developing sucker and pike. The ‘modes are modules’ hypothesis also predicts that genetic or functional genomic studies might well show up independent molecular bases to patterning along the different edges of the Op, and perhaps for other bones as well.

### Cellular Recruitment

Using transgenic lines to mark cells associated with the developing Op provides a cellular basis for the bone growth and shaping patterns we have described. Subsets of mesenchymal cells and their derivatives in the pharyngeal arches are labeled in the two lines we employ for these studies. Among cranial neural crest derivatives, *fli1:eGFP* expressing cells include cartilage, and a variety of other mesenchymal derivatives including osteoblasts and, likely, their precursors (see Materials and Methods). The *osx:eGFP* transgene marks osteoblasts that actively secrete bone as we judge from their positions immediately adjacent to developing bone that we visualize by its mineralization. The only exception to such immediate apposition to the Alizarin Red-stained matrix is along the most rapidly growing vp edge of the Op, where the *osx:eGFP*-labeled population extends out several rows. Only in the first (innermost) of these rows are cells in intimate contact with newly mineralizing matrix, and these inner cells have a distinctive round shape. The outer cells, flattened in shape, could be constantly recruited into the inner layer in a steady state fashion, as growth of the vp edge progresses. Cells expressing *osx:eGFP* that line the jp edge in early larva, when this edge grows only very slowly, also have an elongated and flattened shape. That being so, a flat morphology might indicate relatively low secretory activity, as has been suggested for osteoblasts in tissue culture preparations examined by scanning electron microscopy [43].

Recruitment of osteoblasts and perhaps increase in rates of secretion likely occurs not just at the vp edge showing banding growth, but also at the sites of spur and veil formation that more specifically drive changes in bone shape. Thus, at 11 dpf the *osx:eGFP*-labeling along vj veil and j apex spur shows numerous rounded cells in clusters (Figure 7). In contrast, along the jp edge, very slowly growing at this stage, the cells are sparse in number, and flattened – a situation that changes markedly along the same edge during jp veil formation later. Our studies do not show how the increased numbers of osteoblasts we see locally associated with veils and spurs might arise. The *osx:eGFP*-expressing population is only a subset of the mesenchyme associated with the growing bone, at least at early stages, such that enlisting cells not expressing this marker is a clear possibility, among others including cell division and rearrangement of *osx*-expressing cells, that can be examined in future studies.

## Materials and Methods

### Zebrafish Strains

We used the inbred strain AB zebrafish for most of the experiments, generated at the University of Oregon Zebrafish Facility, and available from the Zebrafish International Resource Center (ZIRC). We also studied two transgenic lines: The *Tg(fli1a:EGFP)y1* line, in which bone-lineage cells appear to fully labeled (see Results section) has been used previously for observations of craniofacial skeletal development ([44]–[46], and unpublished observations), and is available at ZIRC. The cell types expressing the *Tg(fli1a:EGFP)y1* transgene include postmigratory, undifferentiated neural crest-derived cells, chondrocytes, osteoblasts, and mesenchymal cells in cranial ligaments, tendons, and other connective tissues. We recently generated the *Tg(osx:egfp)b1212* line, labeling osteoblasts, in our laboratory by BAC-mediated transgenesis. The line will be deposited at ZIRC, and a full description will be published elsewhere (A. DeLaurier, in preparation).

### Rearing, Husbandry and Staging

Improper husbandry and rearing yields fish mortality, low growth rates and variation among clutches, and for this study we attempted to achieve optimal growth and uniformity within and among clutches throughout many weeks of development. We studied fish generated and kept in our well-run University of Oregon Facility. We obtained embryos from natural spawnings. Fish were reared at 28.5°C with a daily light:dark cycle of 14:10 hr. Embryos were kept in E2 embryo medium [47], at 25 embryos/100 ml, in 100 mm disposable Petri dishes. We staged embryos during the cleavage period to assure a precise estimation of the time of fertilization and rechecked stages during embryogenesis using a standard series [48]. Newly hatched larvae were moved to beakers and E2 replaced with water from the Facility system. We measured standard lengths, defined as the measurement from the anterior-most tip of the jaw to the posterior-most tip of the notochord (early larvae) or hypurals (later) to ascertain growth rates (see also [31]), avoiding unnecessary use of anesthesia in order to limit growth retardation. Similarly, we only used individual fish for single experimental observations of skeletal development (and did not return them to tanks) in order to avoid growth retardation due to stress. We also kept the fish at low density to aid optimal growth. Before 9 dpf we reared the fish in beakers at 15 fish/100 ml, changing 75% of the medium daily, and feeding twice daily after hatching with 12 ml of live paramecia, at 800 paramecia/ml. Between 9 dpf and 21 dpf the sets of 15 fish were reared in 1 liter tanks, and after 21 dpf in 4 l tanks, kept on a Facility-wide recirculating water system. They were fed four times daily with newly hatched brine shrimp naupulii and Ziegler AP100 Larval Diet (<100 µm size early, 250 µm later). Tanks were cleaned daily. Full details of our standard procedures for rearing and husbandry are available from the authors upon request. All of our work with zebrafish has been approved by the University of Oregon Institutional Animal Care and Use Committee (IACUC).

### Bone Staining and Imaging

For most observations we vitally stained mineralized bone in E2 embryo medium containing 50 µg/ml Alizarin Red S (JT Baker catalogue # A475-03) and 10 mM HEPES, pH 7.0. Larvae were stained for 1 to 2 hours in the dark and juveniles (after 21 dpf) were stained overnight in the dark. Fish were rinsed well and for mounting were anesthetized in E2 with 0.017% Tricaine (3-amino benzoic acid ethyl ester, Finquel, from Argent Cat# C-FINQ-UE-5G). The fish were mounted in 0.2% agarose in E2 medium (Ultra-Low gel temperature Type IX Agarose, SIGMA catalogue number A5030) on a drop of 0.3% methyl cellulose in E2 medium between bridged cover slips [47]. After the agarose gelled, the cover slip mount was flooded with the tricaine solution. We imaged preparations with a Zeiss LSM 5 Pascal confocal scanning microscope with AIM software, using a 543 nm excitation laser. We crafted scan settings with attention to capturing the entire opercle in x, y and z planes. To increase resolution, we used a slow scan speed, a very small z slice interval and a small pinhole optimized to 1 airy unit. We used an averaging of 2 to 3 slices. We present image stacks as projections, saved as TIFF files for morphometric analyses.

To observe bone growth between two stages, we labeled larvae with successive pulses of, first, 50 µg/ml Alizarin Red S, and after a period of wash out, a second pulse of 50 µg/ml Calcein (*high purity*, Molecular Probes catalogue # C481, in E2 embryo medium buffered with 1 mM Sodium Phosphate to pH 8.0). We report pulse times in the Figure 4 legend for the individual experiments. We rinsed the fish well, and imaged as above using the 543 nm and the 488 nm lasers and multi track scanning. We used the same settings for two color imaging of Alizarin Red bone staining and eGFP transgenic cell labeling.

For the pulse labeling experiment with the young adult shown in Figure 4, the fish was vitally stained with Alizarin Red S at 100 µg/ml, overnight between 42 and 43 dpf. The stain was washed out and after an 11-day interval of growth in the absence of stain the fish was euthanized at 54 dpf. The Op was dissected out, soft tissue was gently scrubbed away with a wooden toothpick, and the Op was imaged with a Zeiss Axiophot microscope (20x objective).

For the adult bones shown in Figure 8, the zebrafish Op was stained with Alizarin Red S after dissection from a euthanized, unfixed 40 dpf fish and photographed with oblique transmitted illumination to reveal banding and other features of the matrix, including the two struts described in the text. The sucker Op was picked up by one of us on the shore of Chateaugay Lake in New York (USA), and photographed as a dried preparation with flat incidence illumination. The northern pike Op was dissected from a fresh-frozen head, and photographed as for the sucker but after only partial drying to better reveal the banding. The pike head was a gift from Jonathan Gustafson of the Minnesota (USA) Department of Natural Resources, who noted from the image shown in Figure 8 that the fish appeared to be about 5 years old. Hence for the pike the more prominent bands (which are subdivided by narrower bands) are approximately annual, rather than diurnal as we have estimated for zebrafish.

### Geometric Morphometrics

For the morphometrics analyses we used the ‘tps’ software package from the State University of New York at Stony Brook, digitizing the positions of the sixteen landmarks shown in Figure 5D (tps Dig version 2.04 software; [49]). We treated thirteen of these landmarks (those in between j, v, and p) as sliding semi-landmarks [50], reviewed in [51]. From a data set (n = 176), we aligned the configurations by Procrustes generalized least squares superimposition, removing size, rotation, and translation effects. We saved the aligned data, centroid sizes (CS), and the set of principal component (PC) scores (identical to relative warp scores) from each configuration (tps Relative warps version 1.42 software; [52]). Subsets of the aligned configurations were averaged in order to compare the thin plate spline deformations shown in Figure 5D (tpsThin-plate spline version 1.20 software; [53]).
